# Cytotoxicity assessment of exfoliated MoS_2_ using primary human mast cells and the progenitor cell-derived mast cell line LAD2†

**DOI:** 10.1039/d3na00863k

**Published:** 2024-03-29

**Authors:** Hazel Lin, Antonio Esau del Rio Castillo, Viviana Jehová González, Francesco Bonaccorso, Ester Vázquez, Bengt Fadeel, Alberto Bianco

**Affiliations:** a CNRS, Immunology, Immunopathology and Therapeutic Chemistry, UPR 3572, University of Strasbourg, ISIS 67000 Strasbourg France a.bianco@ibmc-cnrs.unistra.fr; b BeDimensional Lungo Torrente Secca 30r Genoa Italy; c Biograph Solutions, Regional Institute of Applied Scientific Research (IRICA), Department of Organic Chemistry, Faculty of Science and Chemistry Technologies, University of Castilla-La Mancha Ciudad Real 13071 Spain; d Nanosafety & Nanomedicine Laboratory, Institute of Environmental Medicine, Karolinska Institutet 177 77 Stockholm Sweden

## Abstract

Molybdenum disulfide is an emerging 2D material with several potential applications in medicine. Therefore, it is crucial to ascertain its biocompatibility. Mast cells are immune cells that are found in many organs and tissues in contact with the extracellular environment, and can be cultured from progenitor cells present in the bone marrow. Given the long period required for differentiation and proliferation of primary mast cells, human mast cell lines have emerged as a tractable model for biological and toxicological studies. Here, we compare two types of industrial MoS_2_ using CD34^+^-derived primary human mast cells and the LAD2 cell line. Minimal effects were observed on early-stage activation endpoints such as β-hexosaminidase release and expression of surface markers of mast cell activation. Transmission electron microscopy revealed limited uptake of the tested materials. Overall, MoS_2_ was found to be biocompatible, and the LAD2 cell line was validated as a useful *in vitro* model of mast cells.

## Abstract 2

Assessment of the impact of molybdenum disulfide on immune cells (*e.g.*, mast cells) is required to ascertain its biocompatibility in view of its future development for biomedical applications.

## Graphical abstract

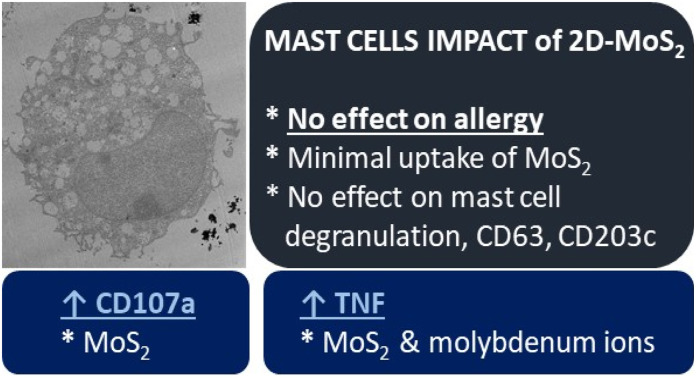

## 1. Introduction

Molybdenum is a widely used industrial component to make alloys, lubricants, and electronics due to its ability to enhance weldability, material strength and corrosion resistance.^1^ Molybdenum is found naturally as oxide or sulfide compounds and is an important micronutrient in plants, animals, and humans although excess exposure has been associated with adverse effects, especially in the case of inhalation exposure to molybdenum trioxide.^2^ Molybdenum has also been implicated in failed orthopedic implants.^3^ Thus, studies have found that molybdenum, as with metal ions in general, can leach from medical devices into patients,^4^ and was found to accumulate in rat liver and kidney cells.^5^ Molybdate ions released from stainless steel stents were also implicated in restenosis (reduction in the diameter of the vessel lumen) in patients bearing coronary artery stents.^6^ However, findings regarding molybdenum safety have been conflicting as no plasma molybdenum ion elevation was observed after a year in patients receiving molybdenum-coated implants compared to standard implants.^7^ A clinical study of implant patients also did not find molybdenum hypersensitivity using the lymphocyte transformation test.^8^

Two-dimensional (2D) molybdenum disulfide, an emerging material in the expanding world of 2D materials, is similar to other 2D transition metal dichalcogenides (TMDs) in that it has unsaturated edge coordination, high surface-volume ratio, and potentially metallic, semi-metallic, or semi-conducting electronic structures.^9^ MoS_2_ nanosheets are the most widely used templates on which to grow other nanomaterials, as recently reviewed.^10^ MoS_2_ in different phases have also been shown to have different physical properties such as stronger near-infrared photoacoustic imaging signals with the 1T phase as compared to the 2H phase.^11^

MoS_2_ can be oxidized into water-soluble molybdate species (*e.g.*, MoO_4_^2−^) and has been found to be less toxic than graphene oxide and its subfamilies^12^ and our previous work has shown that MoS_2_ is susceptible to degradation,^13^ with minimal toxicity evidenced towards primary human macrophages^14,15^ and dendritic cells.^16^ However, other investigators have shown that aggregated forms of MoS_2_ display more cytotoxicity towards lung cells and liver cells than well-dispersed MoS_2_.^17,18^ Moreover, molybdenum ions, derived from MoS_2_ complexed with human serum albumin, were reported to affect molybdenum-dependent enzymes through elemental incorporation.^19^ As MoS_2_ is already under investigation for numerous biomedical applications such as miRNA detection,^20^ hydrogen peroxide sensing,^21^ and drug delivery,^22^ further study to ascertain its biocompatibility is needed.

Mast cells are tissue-resident immune cells that originate from bone marrow progenitor cells.^23^ The name derives from ‘Mastzellen’, meaning well-fed or nutritious cells; the term relates to the fact that these cells are replete with granules.^24^ In fact, mast cells release numerous mediators from intracellular stores including histamine, serotonin, heparin, prostaglandins, leukotrienes, proteases, and cytokines such as TNF-α and IL-4, especially when activated *via* surface receptors (*e.g.*, FceRI or MRGPX2).^25^ Mast cells are found in nearly all tissues, predominantly in tissues exposed to the environment like the gut, lungs, and skin.^26^ Mast cells are, however, challenging to purify from tissues.^27^ Mast cells derived from CD34^+^ cells in peripheral blood are therefore a viable alternative to human bone marrow-derived cells given the ease of obtaining donor blood, with surface receptors such as CD203c and CD63 serving as convenient readouts to identify them.^28^ Moreover, given the relatively high cost and technical difficulty in primary mast cell isolation, mast cell lines that express FceRI surface receptors have been explored as more convenient and cost-effective *in vitro* alternatives.^29,30^ In particular, the Laboratory of Allergic Diseases 2 (LAD2) cell line, established 20 years ago from CD34^+^ cells following marrow aspiration of a patient with mastocytosis with no *KIT* mutations,^29^ has been widely used to study mast cell biology.

Mast cells, along with basophils, are immune cells relevant to material hypersensitivity although heavy metals such as mercury and silver have been traditionally investigated due to their role as non-essential metals.^31^ However, on the one hand, it is relevant to verify if other metals such as molybdenum have similar hypersensitive properties as dental implants containing molybdenum were found to increase rat mast cell count with extensive degranulation.^32^ On the other hand, molybdenum ions (Mo^5+^) did not trigger histamine release in human tissue-derived mast cells.^33^ The latter result highlights the importance of further research on molybdenum hypersensitivity, to gain clearer insight for researchers in the field. Previous studies revealed that silver (Ag) nanoparticles triggered mast cell degranulation.^34,35^ Moreover, short exposures of environmentally relevant metal and transition metal ions such as Al^3+^, Ni^2+^, Cd^2+^ and Sr^2+^ were found to enhance FceRI-mediated mast cell activation. Molybdenum is a transition metal, like cadmium, zinc, copper, cobalt, nickel, mercury and manganese, which have been tested in mast cell lines such as LAD2. CuInS_2_/ZnS–PEG-QDs have been found to enter LAD2 without causing degranulation or IL-8 and TNF release.^36^ In a study involving various metal ions added to mast cells from an assortment of tissues, no effect was observed with transition metals Co^2+^, Cu^2+^, Zn^2+^, Ni^2+^ and Mo^5+^.^37^ The Mo^4+^ ion in MoS_2_ has yet to be tested in mast cells, and we hope our study will add to the literature regarding this metal ion.

Few publications report the effects of 2D materials on primary mast cells or mast cell lines although a previous study disclosed that neither graphene nor graphene oxide triggered histamine, IL-6, or IL-8 release in HMC-1 mast cells.^38^ The present work aimed to investigate the biocompatibility of 2D MoS_2_ nanosheets using both CD34^+^-derived cultured primary human mast cells and the human LAD2 mast cell line. Other mast cell lines, such as HMC-1 and RBL-2H3 have been used in previous studies. However, the RBL-2H3 cell line is derived from basophils and is not fully representative of mast cells^39^ compared to the LAD2 line. HMC-1 cells represent instead immature mast cells while LAD2 represent intermediately differentiated mast cells and are therefore a better alternative.^40^ To this end, we compared two different types of 2H phase industrially produced MoS_2_ flakes prepared according to two different but commonly used methods,^41^ along with molybdenum ions as an experimental control.

## 2. Materials and methods

### 2.1. Preparation of 2D materials and controls

2D MoS_2_ flakes obtained by two different scalable processes were investigated; both materials have been extensively characterized previously.^41^ In brief, for the material obtained from BeDimensional (Italy), designated BS-MoS_2_, crushed MoS_2_ crystals (Smart Elements) were mixed with water and sodium deoxycholate (Sigma-Aldrich) at 0.1 wt% and then exfoliated using a high-pressure piston homogenizer to obtain BD-MoS_2_ flakes.^42^ Sodium deoxycholate (Sigma) was therefore included as an experimental control and used at a similar residual concentration of 30 μg mL^−1^ as per the highest MoS_2_*in vitro* concentration tested. For the material obtained from Biograph Solutions (Spain), designated BS-MoS_2_, bulk MoS_2_ was ball-milled with glycine (all from Sigma-Aldrich) as exfoliating agent, followed by dispersion in water and further dialysis heating to remove excess glycine. Dispersions were lyophilized to BS-MoS_2_ powder, and stored until dispersed in water.^43,44^ For the molybdate ion control, sodium molybdate dihydrate (Sigma) was used at a final concentration of 75 μg mL^−1^ as per the highest MoS_2_*in vitro* concentration tested. The positive control compound 48/80 (C48/80) was purchased from Sigma. All materials tested were endotoxin-free using a previously established macrophage activation assay based on primary human macrophages.^45^ To avoid the possible spontaneous transformation of MoS_2_,^13,46^ dispersions were stored in the dark and under argon. Regular controls were conducted using X-ray photoelectron spectroscopy (XPS) to monitor the eventual oxidation of dispersed materials.

### 2.2. Transmission electron microscopy (TEM)

Cells were fixed with 2.5% glutaraldehyde (Fluka Analytical, Sigma) in 0.1 M phosphate buffer, then post-fixed with 0.5% osmium tetroxide (from EMS) in water and dehydrated through a series of ethanol before being embedded in epoxy resin (Embed 812, EMS). Ultrathin sections (Leica EM UC6) were counterstained with uranyl acetate and observed with a Hitachi 7500 transmission electron microscope (Hitachi High Technologies Corporation, Tokyo, Japan) equipped with an AMT Hamamatsu digital camera (Hamamatsu Photonics, Hamamatsu City, Japan).

### 2.3. Scanning electron microscopy (SEM)

Cells were fixed with 2.5% glutaraldehyde in 0.1 M phosphate buffer for 24 h, then dehydrated through a series of ethanol before being dried with hexamethyldisilazane (Merck, France). The samples were sputter-coated with gold–palladium and observed under a Hitachi S-800 electron microscope.

### 2.4. Isolation and culture of primary mast cells

Human peripheral blood CD34^+^ cells were isolated from buffy coats obtained from the French Blood Bank (Etablissement Français du Sang, Strasbourg, France, contract no. ALC/PIL/DIR/AJR/FO/606). The blood samples were from anonymous healthy donors, therefore making ethical approval unnecessary. CD34^+^ cells were directly isolated from peripheral blood mononuclear cells (PBMCs) using a commercial kit (Miltenyi, #130-100-453). Using a protocol adapted from Arock *et al.*^27^ and Yin *et al.*,^47^ CD34^+^ cells were first left for a week in expansion medium StemSpan™ SFEM II (StemCell, #09605) supplemented with 20 ng mL^−1^ IL-3, 100 ng mL^−1^ IL-6 and 100 ng mL^−1^ SCF (Peprotech). The cells were then differentiated for 8 weeks using IMDM (Lonza, #12-722F) media supplemented with 0.5% BSA, 50 μM β-mercaptoethanol, 1% insulin–transferrin–selenium, 1% penicillin/streptomycin, 100 ng mL^−1^ IL-6 and 100 ng mL^−1^ SCF (Peprotech). The medium was changed weekly *via* hemi-depletion. Mast cells were characterized as the non-debris portion, which was FceRI^+^–CD117^+^ (>80%), and visualized with an optical microscope using acidic toluidine blue (Fig. S1†). Mast cells were sensitized overnight with 100 ng mL^−1^ biotinylated IgE. The next day, excess IgE was washed off and cells were stimulated with 100 ng mL^−1^ streptavidin (supplemented with 100 ng mL^−1^ SCF). Mast cells were exposed to the two MoS_2_ materials at a low dose (5 μg mL^−1^) and a high dose (50 μg mL^−1^) and analyzed 1 or 6 h later.

### 2.5. Maintenance of the human mast cell line

The human LAD2 mast cell line was kindly provided by Professor Dean Metcalfe (NIH Main Campus, Bethesda, MD, USA) and cells were cultured according to Kirshenbaum *et al.*^29^ In short, LAD2 cells were grown in StemPro-34 media (ThermoFisher, #10639011) supplemented with 100 ng mL^−1^ SCF. The medium was changed weekly *via* hemi-depletion. Similar to primary mast cells, LAD2 cells were characterized as the non-debris portion which was FceRI^+^–CD117^+^ (>95%), and visualized with an optical microscope using acidic toluidine blue (Fig. S2†). LAD2 cells were sensitized overnight with 100 ng mL^−1^ biotinylated IgE. The next day, excess IgE was washed off and cells were stimulated with 100 ng mL^−1^ streptavidin (supplemented with 100 ng mL^−1^ SCF). Mast cells were exposed to the two MoS_2_ materials at a low dose (5 μg mL^−1^) and a high dose (50 μg mL^−1^) or to the soluble molybdenum salt (75 μg mL^−1^) as control and analyzed 1 or 6 h later.

### 2.6. Flow cytometric analysis of surface markers

The viability and activation of 18 h MoS_2_-treated cells were assessed using flow cytometry (Beckman Coulter Gallios). The cells were washed with 2% FBS in PBS (Flow Cytometry Staining Buffer, FACS Buffer), then stained with the respective antibody mix at 4 °C for 20 min. The anti-human antibodies used to characterize mast cells or to measure activation were FcεRI-FITC (Biolegend, #334608), CD117-APC (BD, #553356), MRGX2-PE (Biolegend, #359004), CD203c-PerCP/Cyanine5.5 (Biolegend, #324608), CD63-PE (BD, #353004) and CD107a-Alexa 647 (Biolegend, #328612). The viability of cells was analyzed by staining with Fixable Viability Dye (eBioscience FVD-eFluor 780, #65-0865-14). Reactive oxygen species (ROS) production was analyzed by staining with CM-H2DCFDA (Thermo Fisher Science, #C6827) for 30 min at 37 °C. After staining, the cells were washed twice with FACS buffer, then resuspended in fresh FACS buffer and analyzed on the flow cytometer as indicated above.

### 2.7. Enzyme-linked immunosorbent assay (ELISA)

Secretion of the cytokines IL-6 (BD Opt-EIA, #555220), TNF-α (BD Opt-EIA, #555212), IL-8 (BD Opt-EIA, #555244) of cells exposed to MoS_2_ (5 and 50 μg mL^−1^), were assayed with ELISA kits according to the manufacturer's instructions. In short, polyvinyl microtiter 96-well plates (Falcon) were coated overnight at 4 °C with 50 μL per well of purified capture antibodies diluted in coating buffer (carbonate/bicarbonate buffer 0.05 M, pH 9.6). After washing with PBS containing 0.05% Tween (PBS–T), a blocking step was performed by adding 5% FBS in PBS (100 μL per well) for 1 h at room temperature. After washing thrice with PBS–T, 50 μL of culture supernatants from the treated cells were added to the respective wells for 2 h at room temperature, along with a respective series of standards as provided in the kits. The plates were then washed five times with PBS–T. Secondary antibodies as provided in the kit were added together with horseradish peroxidase reagent and incubated for 1 h at room temperature. Then, the plates were washed five times with PBS–T, and the presence of cytokines in the tested supernatants was visualized by adding tetramethylbenzidine in the presence of H_2_O_2_. The resulting absorbance was measured at 450 nm after stopping the reaction with 2 N H_2_SO_4_, after 15 min.

### 2.8. Mast cell degranulation assay

The measurement of mast cell degranulation as previously established by Kuehn *et al.*^48^ used the basis of β-hexosaminidase release as a convenient readout. Briefly, primary mast cells and LAD2 cells were sensitized overnight with 100 ng mL^−1^ biotinylated IgE in the respective media. The next day, excess IgE was washed off and cells were stimulated with 100 ng mL^−1^ streptavidin (supplemented with 100 ng mL^−1^ SCF). Mast cells were exposed to MoS_2_ materials and cultured in HEPES-supplemented HBSS at 37 °C for 1 h to avoid media colour interference, and centrifuged at 450 g, at 4 °C for 5 min. Thereafter, 50 μL aliquots of cell-free supernatant were transferred to 100 μL of *p*-nitrophenyl *N*-acetyl-β-d-glucosamide (PNAG) solution in a new 96-well plate, and the remaining 50 μL of supernatant and lysate was resuspended in 150 μL of 0.1% Triton X-100 solution. 50 μL aliquots of this was transferred to 100 μL of PNAG solution in a new 96-well plate. Both plates were incubated for 90 min at 37 °C then 50 μL 0.4 M glycine buffer was added to stop the reaction and the plates were read at 405 nm with a reference filter at 620 nm. Results were described as % degranulation = 100 × (S/N content)/(S/N + lysate content). PNAG solution was comprised of 35 mg of PNAG (Carl Roth, #4062.1) per 10 mL of citrate buffer (40 mM citric acid and 20 mM Na_2_HPO_4_·7H_2_O at pH 4.5).

### 2.9. Statistical analysis

Experiments were conducted at least three times and the data were processed by GraphPad Prism 7. Results are expressed as mean values ± standard deviation (SD). One-way ANOVA followed by Bonferroni's test was performed to determine the statistical differences among samples *versus* control untreated cells (*, *p* ≤ 0.05; **, *p* ≤ 0.01, ***, *p* ≤ 0.001).

## 3. Results and discussion

### 3.1. Synthesis and characterization of 2D MoS_2_

The two types of 2D MoS_2_ investigated here have also been used in a previous study on basophils.^41^ In the current study, the same batches of MoS_2_ were tested on mast cells. Both MoS_2_ materials were produced using a top-down approach. In the case of BD-MoS_2_, the MoS_2_ was exfoliated into few-layers MoS_2_ using a high-pressure homogeniser, which culminated in the scaling up of crystalline, non-oxidised material at ton-scale. The exfoliation was performed in water and sodium cholate, a bile salt naturally produced in the human body. In the case of BS-MoS_2_, the bulk material was exfoliated, in this case, using glycine as an exfoliating agent in a ball milling treatment. The solid phase mechanochemical process is environmentally friendly, cost-effective, and very simple. After exfoliation, glycine could be recovered by water dialysis and reused in subsequent treatments. Only traces of this natural amino acid remain in the final solid material, making this material very useful for bio-applications. The two materials were dispersed in MilliQ® water (1 mg mL^−1^), before use in the cellular experiments. These samples were fully characterized using a combination of complementary microscopic and spectroscopic techniques (full details and related figures are reported in Lin *et al.*^41^). In summary, the morphology (single and few-layers), the lateral size (BD-MoS_2_: 100–850 nm; BS-MoS_2_: 25–375 nm) and the thickness (BD-MoS_2_: 1.2 nm; BS-MoS_2_: 3.2 nm) were obtained by TEM and AFM (Fig. S3†).^41^ Thermogravimetric analysis (TGA) was performed to quantify the amount of sodium cholate (50%) and glycine (3%) in BD-MoS_2_ and BS-MoS_2_, respectively.^41^ The negative zeta potential values, corresponding to −45.86 mV for BD-MoS_2_ and −31.41 mV for BS-MoS_2_, are indicative of a good colloidal stability.^41^ Raman spectra for both materials were characterized by the typical bands of exfoliated MoS_2_ around 380 and 405 cm^−1^.^41^ Finally X-ray photoelectron spectroscopy (XPS) confirmed the oxidation state of molybdenum, corresponding to Mo^4+^, with a negligible presence of oxidized Mo^6+^ species.^41^

### 3.2. Primary mast cell viability and activation

**Fig. 1:**
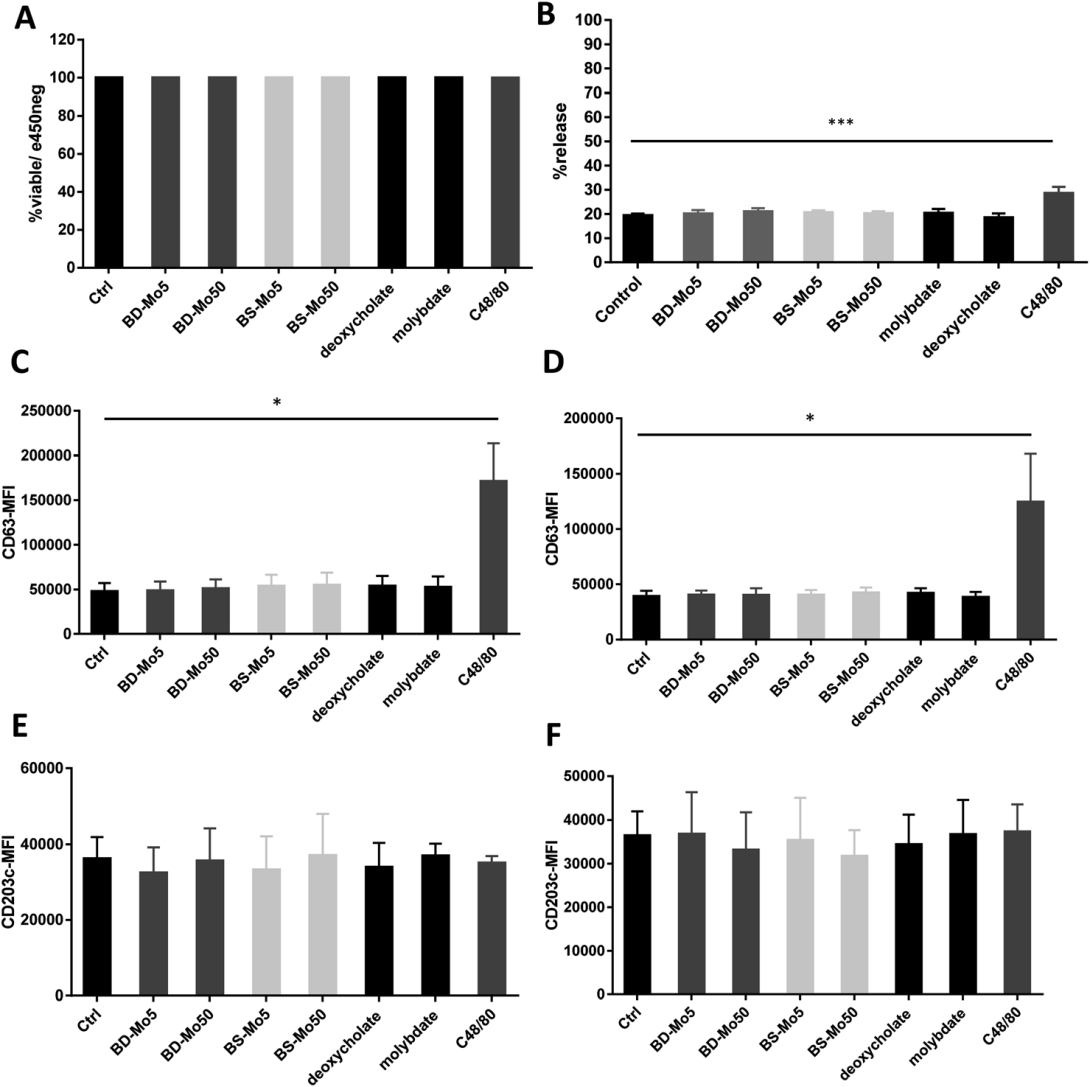
MoS_2_ did not have detrimental impact on mast cell viability and surface markers. Primary mast cells treated with 5, 50 μg mL^−1^ BD- or BS-MoS_2_ for (A) 6 h, viability (B) 1 h, degranulation. CD63 expression of mast cells treated with 5, 50 μg mL^−1^ BD- or BS-MoS_2_ for (C) 1 h (D) 6 h. CD203c expression of mast cells treated with 5, 50 μg mL^−1^ BD- or BS-MoS_2_ for (E) 1 h (F) 6 h. All experiments were conducted thrice in triplicate and shown as mean ± SD. *P < 0.05; **P < 0.01, ***P < 0.001 by one-way ANOVA with Bonferroni post-tests.

Mast cells were developed from CD34^+^ cells isolated from healthy human donor peripheral blood mononuclear cells using a commercial kit as detailed in the Materials and methods section. No difference in viability was seen after 1 h of treatment with the two types of MoS_2_, even with C48/80 (widely used for non-IgE-dependent stimulation of mast cells) at 10 μg mL^−1^ (Fig. 1A).

Degranulation, a process peculiar to mast cells and basophils, involves the release of granules containing pre-formed mediators such as histamine, β-hexosaminidase, or tryptase, with the initiation and strength of the response depending on specific stimuli.^49^ In our study, no difference in mast cell degranulation as measured by β-hexosaminidase release was observed, except with the positive control C48/80 (Fig. 1B). This was not unexpected, given that previous research did not identify molybdenum-containing nanoparticles among a list of metal nanoparticles that could modulate mast cell function.^24^ However, this is the first study to evaluate 2D MoS_2_ nanosheets with respect to the degranulation of mast cells.

CD63 and CD203c have been recognized as suitable markers of mast cell activation.^50^ A crucial component of allergic inflammation, CD63 is expressed on mast cell surfaces and in lysosomes and is required for efficient IgE-mediated mast cell degranulation.^51^ Activated mast cells release ATP thereby triggering purinergic signaling. Upregulated CD203c suppresses this potentially chronic inflammatory response by hydrolysis of extracellular ATP. As marker for allergen sensitivity, CD203c is also expressed in multiple organs, and on epithelial and mucosal surfaces.^52^ Mast cells were exposed to the two types of MoS_2_ and found no change in the activation markers CD63 and CD203c with all concentrations of the different materials tested at 1 h and 6 h. However, the positive control C48/80 significantly affected CD63 (Fig. 1C–F). The mode of action of CD203c has only been found relevant in chronic but not acute inflammatory responses, which could explain why no response was seen even with the positive control C48/80.^52^

**Fig. 2:**
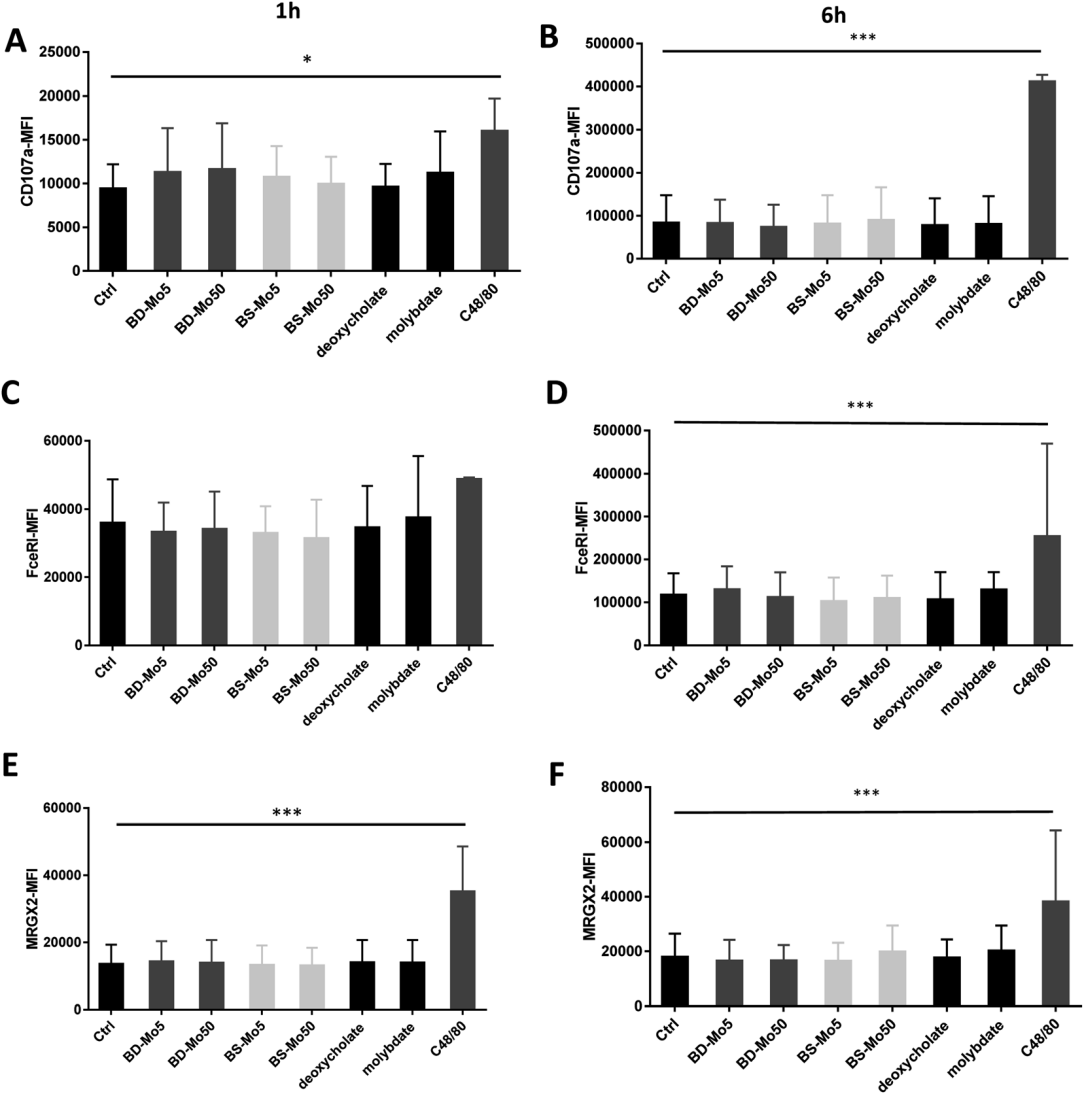
MoS_2_ did not have detrimental impact on mast cell activation markers. CD107a expression of mast cells treated with 5, 50 μg mL^−1^ BD- or BS-MoS_2_ for (A) 1 h (B) 6 h. FceRI expression of mast cells treated with 5, 50 μg mL^−1^ BD- or BS-MoS_2_ for (C) 1 h (D) 6 h. MRGPX2 expression of mast cells treated with 5, 50 μg mL^−1^ BD- or BS-MoS_2_ for (E) 1 h (F) 6 h. All experiments were conducted thrice in triplicate and shown as mean ± SD. *P < 0.05; **P < 0.01, ***P < 0.001 by one-way ANOVA with Bonferroni post-tests.

CD107a is another established mast cell surface activation marker already validated in human blood-derived mast cells.^53^ A non-significant increase of CD107a was observed at 1 h with molybdate and all concentrations of both industrial BD-MoS_2_ and BS-MoS_2_, but not as highly expressed at the positive control C48/80, while CD107a expression at 6 h had higher baseline expression, and was even more pronounced with the positive control (Fig. 2A and B).

The expression of the mast cell IgE receptor FceRI was increased compared to control only with molybdate, with a stronger increase with the positive control C48/80 at both 1 h and 6 h (Fig. 2C and D). The non-IgE-dependent surface receptor MRGX2 demonstrated a non-significant increase with molybdate and high-dose BS-MoS_2_ at 6 h, and with C48/80 at both 1 h and 6 h (Fig. 2E and F). FceRI is also expressed on subsets of myeloid cells related to allergy such as Langerhans cells^54^ and atopic monocytes^55^ while MRGX2 is also expressed in the central nervous system.^56^ This raises the possibility of molybdate eliciting responses in cells other than mast cells, which express FceRI or MRGX2.

### 3.3. Human mast cell line viability and activation

**Fig. 3:**
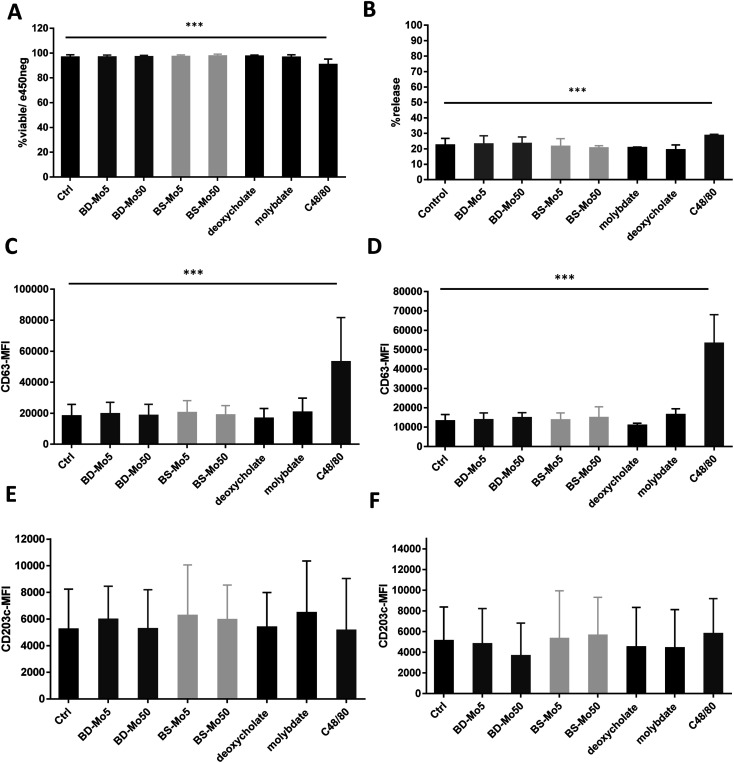
MoS_2_ did not have detrimental impact on LAD2 cell viability and surface markers. LAD2 cells treated with 5, 50 μg mL^−1^ BD- or BS-MoS_2_ for (A) 6 h, viability (B) 1 h, degranulation. CD63 expression of LAD2 cells treated with 5, 50 μg mL^−1^ BD- or BS-MoS_2_ for (C) 1 h (D) 6 h. CD203c expression of mast cells treated with 5, 50 μg mL^−1^ BD- or BS-MoS_2_ for (E) 1 h (F) 6 h. All experiments were conducted thrice in triplicate and shown as mean ± SD. *P < 0.05; **P < 0.01, ***P < 0.001 by one-way ANOVA with Bonferroni post-tests.

LAD2 mast cells were exposed similarly to the CD34^+^-derived mast cells as detailed above. Viability at 6 h was decreased only with the positive control C48/80, with a much lower concentration of 0.1 μg mL^−1^ used in LAD2 cells to obtain an acceptable viability of 85% (Fig. 3A). No difference in mast cell degranulation as measured by β-hexosaminidase release was also observed, except with the positive control C48/80 (Fig. 3B). At 1 h, the expression of the mast cell activation marker CD63 was non-significantly increased with molybdate while at 6 h, a non-significant increase was seen also with high-dose of BD-MoS_2_ and BS-MoS_2_. The positive control C48/80 was clearly heightened at both time points (Fig. 3C and D). No change in CD203c expression at both 1 h and 6 h was seen, even with the positive control C48/80, similar to the primary mast cells as already discussed earlier (Fig. 3E and F).

**Fig. 4:**
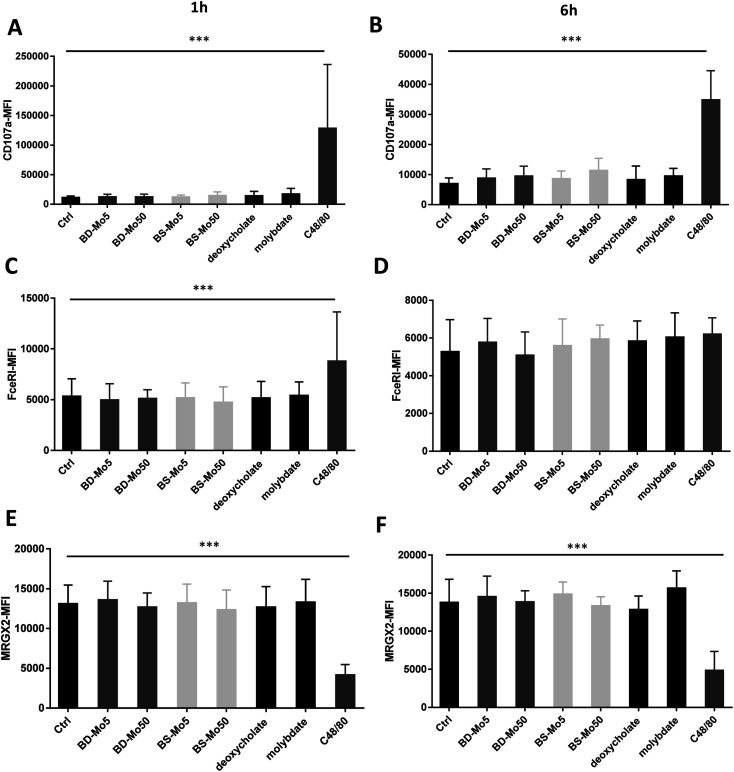
MoS_2_ did not have detrimental impact on LAD2 cell activation markers. CD107a expression of LAD2 cells treated with 5, 50 μg mL^−1^ BD- or BS-MoS_2_ for (A) 1 h (B) 6 h. FceRI expression of mast cells treated with 5, 50 μg mL^−1^ BD- or BS-MoS_2_ for (C) 1 h (D) 6 h. MRGPX2 expression of mast cells treated with 5, 50 μg mL^−1^ BD- or BS-MoS_2_ for (E) 1 h (F) 6 h. All experiments were conducted thrice in triplicate and shown as mean ± SD. *P < 0.05; **P < 0.01, ***P < 0.001 by one-way ANOVA with Bonferroni post-tests.

No change in CD107a expression was seen at 1 h in LAD2 cells except with the positive control (Fig. 4A). However, a non-significant but dose-dependent increase at 6 h was observed with both MoS_2_ and molybdate (Fig. 4B). C48/80 showed a greater increase in CD107a in LAD2 at 1 h compared to primary mast cells. Higher baseline expression of CD107a in human CD34^+^-derived mast cells compared to LAD2 cells was also previously noted by other groups.^57^ Given that CD107a is a measure of late-stage activation and was increased both in primary mast cells and LAD2 cells by molybdate and both MoS_2_, this raises the possibility that molybdate ions are triggering some upstream pathways that could be further investigated.

Increased expression of the mast cell IgE receptor FceRI was noted at 1 h with C48/80 but not at 6 h (Fig. 4C and D) demonstrating the lower sensitivity in LAD2 cells at 6 h compared to primary mast cells. C48/80 unexpectedly produced a decrease of MRGX2 at both time points in contrast to the primary mast cells, with molybdate showing a non-significant increase at 6 h (Fig. 4E and F). LAD2 are known to express MRGX2 at lower levels compared to primary cells. Mast cell activation based on C48/80 administration has been shown to reach a plateau in the LAD2 cell line while it continued to rise sharply in primary mast cells.^58^

### 3.4. Mast cell cytokine secretion and ROS production

**Fig. 5:**
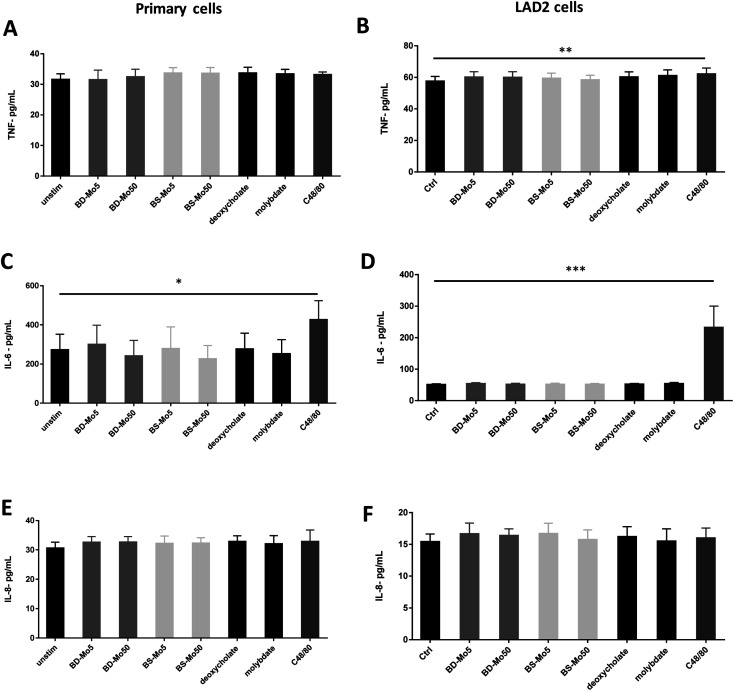
MoS_2_ did not have detrimental impact on mast cell cytokines. TNF-α production of (A) primary mast cells (B) LAD2 cells treated with 5, 50 μg mL^−1^ BD- or BS-MoS_2_ for 6 h. IL-6 production of (C) primary mast cells (D) LAD2 cells treated with 5, 50 μg mL^−1^ BD- or BS-MoS_2_ for 6 h. IL-8 production of (E) primary mast cells (F) LAD2 cells treated with 5, 50 μg mL^−1^ BD- or BS-MoS_2_ for 6 h. All experiments were conducted thrice in triplicate and shown as mean ± SD. *P < 0.05; **P < 0.01, ***P < 0.001 by one-way ANOVA with Bonferroni post-tests.

Mast cell released TNF-α can rapidly initiate neutrophil infiltration upon inflammatory insult,^59^ which can result in a downstream tissue damage. In our experiments, a non-significant increase of the inflammatory cytokine TNF-α was observed at 6 h with BS-MoS_2_, deoxycholate, molybdate and the positive control C48/80 in primary cells (Fig. 5A). TNF-α was however significantly increased at 6 h with the positive control in LAD2 cells, with a non-significant increase with both doses of BD-MoS_2_, deoxycholate and molybdate (Fig. 5B). A more than 5-fold increase in TNF-α production of LAD2 compared to primary mast cells with a different positive control (*e.g.*, IL-33) was also observed in another study,^60^ which makes this a useful readout.

Basophil and mast cell-derived IL-6 have been implicated in allergic and other non-specific inflammatory responses.^61^ IL-6 itself is also crucial in primary human mast cell proliferation and response.^62^ An increase in the inflammatory cytokine IL-6 was only seen with the positive control C48/80 in both primary mast cells and the cell line (Fig. 5C and D). This indicates a lack of inflammatory effect from the tested materials, thus expanding our knowledge on immune cell interactions of MoS_2_.^14,16^

IL-8 is another cytokine produced by mast cells, which contributes to neutrophil recruitment.^63^ This cytokine has been identified in inflammatory diseases such as rheumatoid arthritis, psoriasis and lung diseases and has been established to also be inducibly produced by mast cell lines such as HMC-1.^64^ Our results however showed no augmented response of IL-8 even with C48/80 in both primary mast cells and LAD2 cells (Fig. 5E and F). This could be due to differences in the measurement methods as many studies used PCR, which quantified mRNA as opposed to our ELISA method, which focused on actual protein secretion.

Mast cells produce ROS intracellularly, and ROS production was found to be functionally linked to mast cell activation.^65^ No differences in ROS production at 1 h for any of the materials and controls for primary mast cells were observed (Fig. S4A†). With respect to the LAD2 cell line, molybdate prompted a slight increase in ROS production compared to untreated control while this was more pronounced for deoxycholate (Fig. S4B†).

### 3.5. Electron microscopy of material interactions with mast cells

**Fig. 6:**
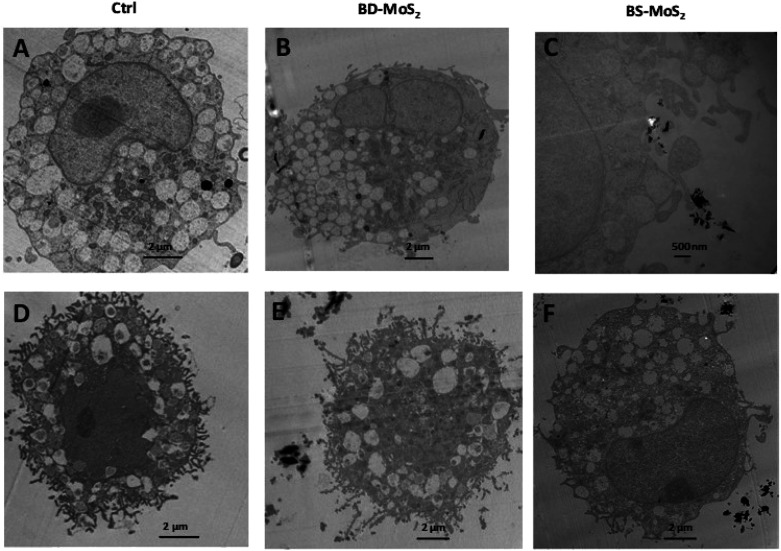
MoS_2_ uptake or interaction with LAD2 cells and primary mast cells. TEM images of LAD2 cells (A) untreated or (B) treated with 50 μg mL^−1^ BD-MoS_2_ for 1 h, or (C) treated with 50 μg mL^−1^ BS-MoS_2_ for 1 h. TEM images of primary mast cells (D) untreated (E) treated with 50 μg mL^−1^ BD-MoS_2_ for 1 h, (F) treated with 50 μg mL^−1^ BS-MoS_2_ for 1 h.

The acquisition of TEM images of 1 h-exposed mast cells was challenging due to the fragility of the cells. This was the case, especially for primary mast cells. Notwithstanding, in general, the presence of the darker-coloured MoS_2_ led to lower image contrast for intracellular organelles, as previously seen for macrophages.^14^ Compared to untreated control LAD2 cells (Fig. 6A), most material was found outside the cells (Fig. 6B). Small quantities of BD-MoS_2_ were observed within vacuolar components in a few LAD2 cells and this was not deemed representative. This was not surprising given that mast cells are known to phagocytose foreign material such as pathogens but far from the extent of macrophages.^66^ BS-MoS_2_ was noted to be associated with the surface of LAD2 mast cells (Fig. 6C). For primary mast cells, both types of MoS_2_ were found outside the cell (Fig. 6D–F). This implied that the cellular impact of MoS_2_ observed in previous assays did not require uptake but most likely cell surface contact. Previous work has shown that graphene oxide sheets can orient parallel to the cell membrane^67^ and this ‘masking’ could also play a role for subsequent biological effects.

Scanning electron microscopy was also performed on the LAD2 cell line which is sturdier than primary mast cells. Cells were exposed for 1 h with both MoS_2_. Only a small portion of the material for both types of MoS_2_ was found in contact with LAD2 cells based on SEM images compared to untreated control (Fig. S5A–C†), which supported the TEM data in that almost no materials were taken up.

## 4. Conclusions

Mast cells are an important immune cell type with key roles in allergic inflammation. Using a battery of assays comparing two types of industrially relevant MoS_2_ in both primary human mast cells and the human LAD2 mast cell line, we found that both MoS_2_ materials are generally biocompatible. Only the non-acute phase marker CD107a showed an increase, but this was also seen for the molybdate ion control, therefore not being a 2D material-specific response. Moreover, using electron microscopy, almost no material was observed to be taken up by primary mast cells or the LAD2 mast cell line. The fact that most early-stage activation readouts in this *in vitro* studies such as the β-hexosaminidase release assay, and CD63/CD203c expression were not affected by both tested materials, suggests that MoS_2_ is unlikely to cause acute (mast cell-dependent) allergic inflammation. Non-significant increases were observed for TNF-α both in primary cells and LAD2 cells exposed to MoS_2_, which makes this cytokine a relevant choice in future screening assays. The LAD2 cell line displayed lower expression levels for multiple markers; however, these cells were found to be a viable *in vitro* option for 2D material testing, being less expensive, less time-consuming, and less laborious than primary cells.

There is a pressing need to consider the role of environmental factors in allergic diseases such as air pollution given that IgE-mediated allergic inflammation is a key pathological mechanism. Mast cells are critical effector cells present in tissues exposed to the external environment and are capable of releasing various inflammatory mediators. It is important to investigate the possible impact of metal ions or metal based as this may shed light on the potential exacerbation of allergic diseases. Indeed, although we addressed the impact of MoS_2_ nanosheets on mast cells from healthy individuals, it remains equally crucial in future studies to consider the population of susceptible individuals who have or who are at risk of developing allergic diseases.

## Author contributions

H. L. designed, performed and analysed the biological experiments, and wrote the first draft; A. E. D. R. C., V. J. G., and L. J. prepared and characterized the materials; B. F. contributed to the discussion of the results; E. V. supervised the preparation of the materials; A. B. led the study and supervised the work. All co-authors read, commented on, and edited the manuscript.

## Conflicts of interest

The authors declare no conflicts of interest.

## Supplementary Material

NA-006-D3NA00863K-s001
