# Supplementary material for: Cytotoxicity assessment of exfoliated MoS2 using primary human mast cells and the progenitor cell-derived mast cell line LAD2
Source: Nanoscale Adv. 2024 Mar 29;6(9):2419–30. doi: 10.1039/d3na00863k (PMC11059565; doi:10.1039/d3na00863k)
Supplement: NA-006-D3NA00863K-s001 [file NA-006-D3NA00863K-s001.pdf]

Electronic Supplementary Material (ESI) for Nanoscale Advances.
This journal is © The Royal Society of Chemistry 2024

Cytotoxicity assessment of exfoliated MoS_2 using primary human mast cells and the progenitor cell-derived mast cell line LAD2†

Hazel Lin,[1] Antonio Esau del Rio Castillo,[2] Viviana Jehová González,[3] Francesco Bonaccorso,[2] Ester Vázquez,[3] Bengt Fadeel,[4] Alberto Bianco[1,*]

[1]CNRS, Immunology, Immunopathology and Therapeutic Chemistry, UPR 3572, University of Strasbourg, ISIS, 67000 Strasbourg, France

[2]BeDimensional, Lungo Torrente Secca 30r, Genoa, Italy

[3]Biograph Solutions, Regional Institute of Applied Scientific Research (IRICA), Department of Organic Chemistry, Faculty of Science and Chemistry Technologies, University of Castilla-La Mancha, Ciudad Real 13071, Spain

[4]Nanosafety & Nanomedicine Laboratory, Institute of Environmental Medicine, Karolinska Institutet, 177 77 Stockholm, Sweden

ELECTRONIC SUPPLEMENTARY INFORMATION

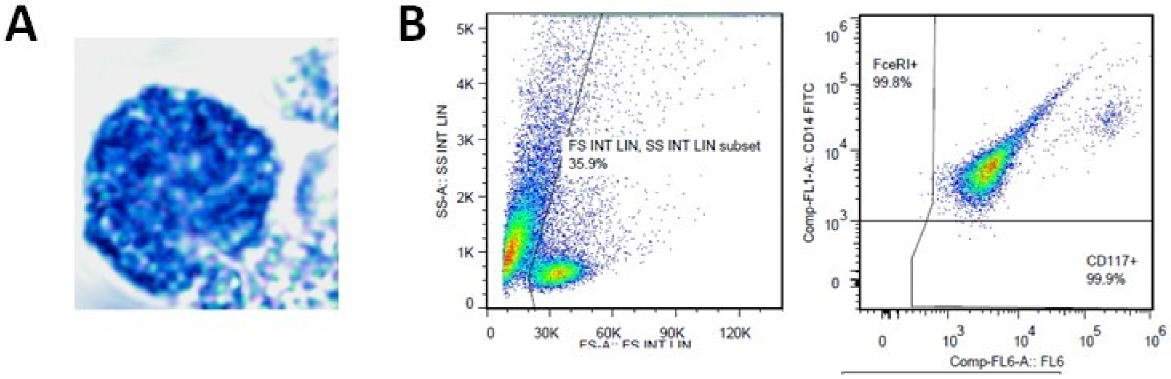

Figure S1. Characterization of primary mast cells. Optical microscopy images of primary mast cells (A) stained with acidic toluidine blue. Flow cytometry characterization of primary mast cells (B) stained with FceRI and CD117.

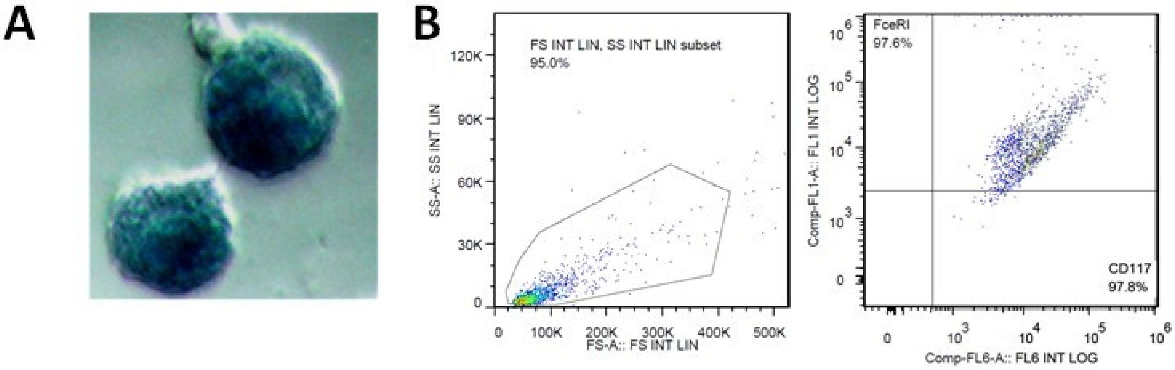

Figure S2. Characterization of LAD2 cells. Optical microscopy images of primary mast cells (A) stained with acidic toluidine blue. Flow cytometry characterization of LAD2 cells (B) stained with FceRI and CD117.

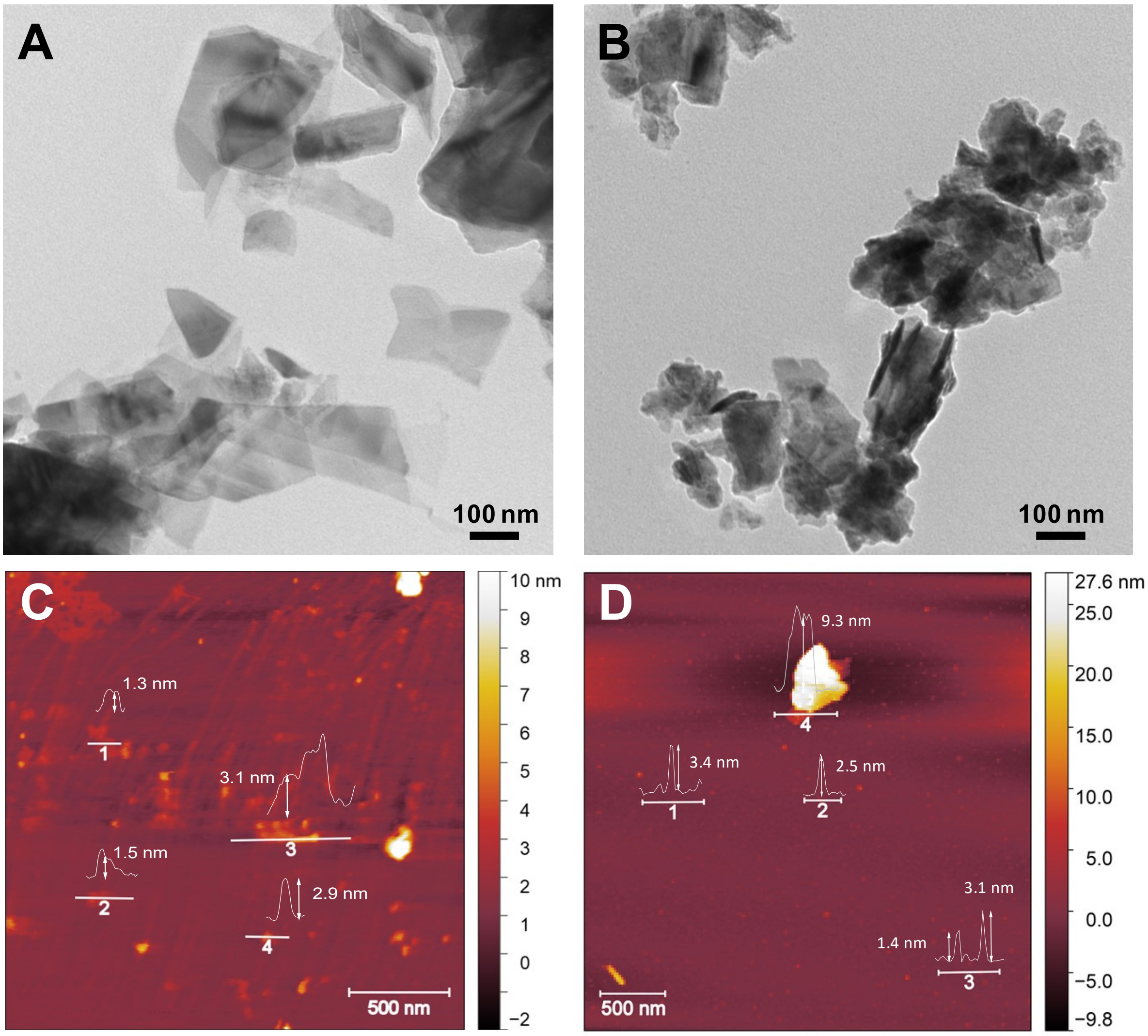

Figure S3. TEM and AFM analysis of MoS_2. (A) TEM image of selected BD-MoS_2 flakes; (B) TEM image of selected BS-MoS_2 flakes; (C) AFM image of selected BD-MoS_2 flakes; (D) AFM image of selected BD-MoS_2 flakes.

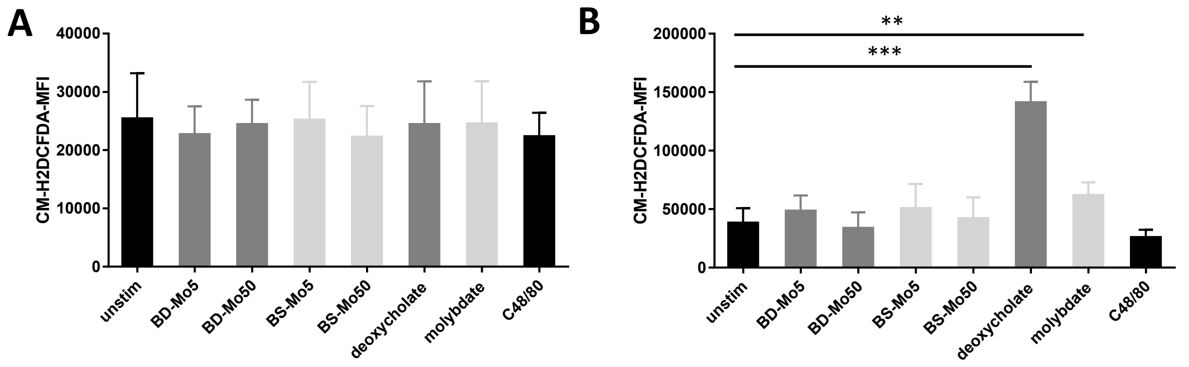

Figure S4. ROS production in primary mast cells and LAD2 cells. ROS production of **(A)** primary mast cells **(B)** LAD2 cells treated with 5, 50 µg mL^{-1} BD- or BS-MoS_2 for 1h and stained with CM-H2DCFDA. All experiments were conducted thrice in triplicate and shown as mean ± SD. *P<0.05; **P<0.01, ***P<0.001 by one-way ANOVA with Bonferroni post-tests.

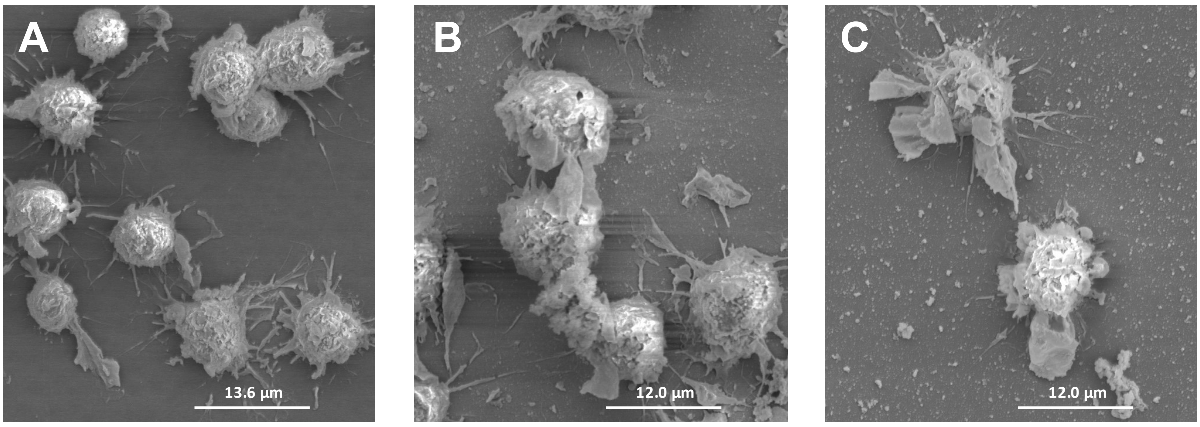

Figure S5. SEM of unexposed and exposed LAD2 cells. (A) Untreated LAD2 cells. LAD2 cells treated with 50 µg mL^{-1} **(B)** BD-MoS_2 or **(C)** BS-MoS_2 for 1h.
